# Integrative Genomic and in Silico Analysis Reveals Mitochondrially Encoded Cytochrome C Oxidase III (MT—CO3) Overexpression and Potential Neem-Derived Inhibitors in Breast Cancer

**DOI:** 10.3390/genes16050546

**Published:** 2025-04-30

**Authors:** Oluwaseun E. Agboola, Samuel S. Agboola, Oluwatoyin M. Oyinloye, Abimbola E. Fadugba, Esther Y. Omolayo, Zainab A. Ayinla, Foluso O. Osunsanmi, Oluranti E. Olaiya, Folake O. Olojo, Basiru O. Ajiboye, Babatunji E. Oyinloye

**Affiliations:** 1 Institute for Drug Research and Development, Bogoro Research Centre, Afe Babalola University, Ado-Ekiti 360001, Nigeria; agboolaoe@abuad.edu.ng; 2 Damsem Scientific Laboratory and Research, Ado-Ekiti 360102, Nigeria; 3 Department of Pharmacology and Toxicology, College of Pharmacy, Afe Babalola University, Ado-Ekiti 360001, Nigeria; agboolass@abuad.edu.ng; 4 Department of Biological Sciences, College of Sciences, Afe Babalola University, P.M.B 5454, Ado-Ekiti 360001, Nigeriafadugbaae@abuad.edu.ng (A.E.F.); omolayoesther@gmail.com (E.Y.O.); 5 Department of Biology, University of Waterloo, Waterloo, ON N2L 3G1, Canada; zayinla@uwaterloo.ca; 6 Biotechnology and Structural Biology (BSB) Group, Department of Biochemistry and Microbiology, University of Zululand, KwaDlangezwa 3886, South Africa; alafin21@yahoo.com; 7 Department of Medical Biochemistry, College of Medicine and Health Sciences, Afe Babalola University, Ado-Ekiti 360001, Nigeria; olaiyaoe@abuad.edu.ng; 8 Department of Chemical Sciences, Dominion University, Ibadan 110108, Nigeria; f.olojo@dominionuniversity.edu.ng; 9 Phytomedicine and Molecular Toxicology Research Laboratory, Department of Biochemistry, Federal University Oye-Ekiti, Oye-Ekiti 371104, Nigeria; bash1428@yahoo.co.uk; 10 Phytomedicine, Biochemical Toxicology and Biotechnology Research Laboratories, Department of Biochemistry, College of Sciences, Afe Babalola University, P.M.B 5454, Ado-Ekiti 360001, Nigeria

**Keywords:** MT—CO3, breast cancer, neem compounds, molecular docking, mitochondrial therapeutics

## Abstract

Background: The increasing global incidence of breast cancer calls for the identification of new therapeutic targets and the assessment of possible neem-derived inhibitors by means of computational modeling and integrated genomic research. Methods: Originally looking at 59,424 genes throughout 42 samples, we investigated gene expression data from The Cancer Genome Atlas—Breast Cancer (TCGA-BRCA) dataset. We chose 286 genes for thorough investigation following strict screening for consistent expression. R’s limma package was used in differential expression analysis. The leading candidate’s protein modeling was done with Swiss-ADME and Discovery Studio. Molecular docking studies, including 132 neem compounds, were conducted utilizing AutoDock Vina. Results: Among the 286 examined, mitochondrially encoded cytochrome C oxidase III (MT—CO3) turned out to be the most greatly overexpressed gene, showing consistent elevation across all breast cancer samples. Protein modeling revealed a substantial hydrophobic pocket (volume: 627.3 Å^3^) inside the structure of MT—CO3. Docking investigations showed five interesting neem-derived inhibitors: 7-benzoylnimbocinol, nimolicinol, melianodiol, isonimocinolide, and stigmasterol. Strong binding affinities ranging from −9.2 to −11.5 kcal/mol and diverse interactions with MT—CO3, mostly involving the residues Phe214, Arg221, and Trp58, these molecules displayed. With hydrophobic interactions dominant across all chemicals, fragment contribution analysis revealed that scaffold percentage greatly influences binding effectiveness. Stigmasterol revealed greater drug-likeness (QED = 0.79) despite minimal interaction variety, while 7-benzoylnimbocinol presented the best-balanced physicochemical profile. Conclusion: Connecting traditional medicine with current genomics and computational biology, this work proposes a methodology for structure-guided drug design and development using neem-derived chemicals and finds MT—CO3 as a potential therapeutic target for breast cancer.

## 1. Introduction

Breast cancer is a significant global health problem, and it has been estimated that 2.3 million new cases are diagnosed every year [1]. Even with major improvements in finding and treating breast cancer early, the variety and differences in breast cancer cells make it difficult to create effective long-term treatments [2]. Recently, there has been a major change in cancer research, recognizing that problems with mitochondria play a key role in how cancer starts and grows [3]. The mitochondrial genome, previously underappreciated in cancer biology, has emerged as an important factor in cellular bioenergetics and neoplastic transformation [4]. Of particular interest is the MT—CO3 gene that encodes cytochrome c oxidase subunit III (complex IV) of the mitochondrial electron transport chain. While nuclear-encoded mitochondrial genes have been extensively studied, the function of MT—CO3 in initiating breast cancer pathogenesis is not well studied [5]. Concurrently, the renewed interest is being directed to the discovery of the enormous drug discovery of natural products as a treasure trove of novel anticancer agents [6]. The neem tree (*Azadirachta indica*), whose vast wealth of health values has been prized since time immemorial, has been reported to exhibit vigorous anticancer activity in different preclinical models [7]. Various compounds have been found in earlier research to act as inhibitors of MT—CO3 activity, such as cyanide derivatives, carbon monoxide, and certain antimalarial agents like atovaquone that target the electron transport chain [5,8]. Biguanides such as metformin specifically have been found to inhibit the activity of complex IV, which comprises the MT—CO3 subunit, leading to cancer cell metabolic reprogramming [9]. While neem compounds show promising anticancer activity, their potential to interact with off-target proteins must be noted. The identified compounds (7-benzoylnimbocinol, nimolicinol, melianodiol, isonimocinolide, and stigmasterol) have been reported to interact with various cellular targets. For example, stigmasterol has formed interactions with nuclear hormone receptors as well as membrane lipid structures [6,7]. Neem terpenoids such as nimolicinol and melianodiol have shown affinity for several enzymes, including cytochrome P450s and protein kinases involved in cell signaling pathways [10]. Knowledge of these off-target interactions is important to understand the broader biological effects and possible side effects of these compounds when viewed as therapeutic agents targeting MT—CO3. However, the molecular mechanism of its actions, with their potential interactions with mitochondrial targets in breast cancer cells, is as yet not fully elucidated. These critical knowledge gaps will be explored in this research through a pioneering, integrative approach combining state-of-the-art genomics and high-performance computational simulation to conduct an exhaustive analysis of gene expression profiles in breast cancer with particular attention to mitochondrial-encoded genes. Besides, a structural model construct of the MT—CO3 will be made to fill the current structural deficiency of information on mitochondrially encoded proteins. In addition, the study will discover and characterize putative neem-derived molecules that could affect MT—CO3 activity and apply our structural information to perform rational, structure-guided drug discovery. Through these multi-disciplinary strategies combined, we aim to reveal the complex relationship between mitochondrial failure, i.e., MT—CO3 overexpression, and breast cancer tumorigenesis. This research will establish a new paradigm for the targeting of mitochondrial-encoded proteins with natural product-derived compounds, which could open up new lines of breast cancer therapy and result in a new generation of mitochondria-targeted therapeutics against breast cancer.

## 2. Materials and Methods

### 2.1. Data Collection and Preprocessing: TCGA-BRCA Data Retrieval

The data for the Cancer Genome Atlas Breast Invasive Carcinoma (TCGA-BRCA) was retrieved via the Genomic Data Commons (GDC) Application Programming Interface (API) [11]. To allow for more extensive analysis, we collected gene expression quantification data, aligned reads, and clinical supplemental data.

### 2.2. Normalization and Data Processing of RNA Sequencing

RNA sequencing (RNA-Seq) data were computed to achieve a gene expression matrix. HTSeq count files were merged, and gene identifiers were made consistent by removing version numbers from Ensembl IDs. The transcripts per million (TPM) method was used to eliminate length bias introduced by gene length and depth of sequencing to normalize gene expression data [12].

### 2.3. Gene Expression Analysis and Differential Expression

After normalization [13], differential gene expression analysis was performed with R’s limma package with *p* < 0.05 significance cutoff and Benjamini–Hochberg false discovery rate (FDR) multiple testing adjustment. The analysis utilized 113 TCGA-BRCA paired tumor-normal samples, enabling direct within-patient comparison that minimizes individual variation confounding variables. Genes with log2FC > 1.5 were considered significantly different. Expression levels are represented as transcripts per million (TPM) values

### 2.4. Identification of MT—CO3 as a Dominant Overexpressed Gene and Modeling of the MT—CO3 Protein

Mitochondrially encoded cytochrome C oxidase III, or MT—CO3, was identified as the leading over-expressed gene among the differentially expressed genes. Its fold change size and statistical significance were the reasons for this. Since there is little structural information on MT—CO3, we used a protein modeling technique to create a three-dimensional structure for further analysis.

### 2.5. Retrieval and Translation of Sequences

The human MT—CO3 gene genomic DNA sequence (ENSG00000198938) was retrieved from the Ensembl database (version 109). The entire coding sequence was translated into its corresponding amino acid sequence using the ExPASy Translate program (https://web.expasy.org/translate/) (accessed on 3 October 2024) and the vertebrate mitochondrial genetic code. The longest open reading frame was chosen, and a 261-amino acid protein sequence was derived for further structural analysis [14].

### 2.6. Model Validation and Structure Prediction

SWISS-MODEL (version 2024.1) was used for homology modeling [15]. A BLAST (BLAST+ 2.14.1) search was used to identify the initial template, bovine cardiac cytochrome c oxidase (PDB ID: 1V54, chain C, resolution: 1.8 Å, 87.3% sequence identity) from the PDB database. Human cytochrome c oxidase (PDB ID: 5Z62, resolution: 3.6 Å) was used as a second template for loop regions with poor sequence conservation. The original model was re-folded via 5000 steps of energy minimization in the YASARA2 force field and at a 7.86 Å non-bonded interaction threshold. Quality validation was by ERRAT (89.7 score higher than the 80% mark for high-quality structures) and VERIFY3D (91.8% of residues with ≥0.2 scores, above the 80% quality threshold).

### 2.7. Analysis and Forecasting via Computation

To enable an in-depth structural study and binding site prediction, the MT—CO3 structure was docked on Discovery Studio [16]. The Ramachandran plot, which graphs the distribution of protein backbone phi and psi angles for assessing the stereochemical quality of the model; the hydrophobicity plot, aids in identifying the hydrophobic and hydrophilic regions of the protein and, thus, gives an insight into the possible ligand interactions; and the 3D point plot, which is utilized to graphically represent the spatial distribution of various physicochemical properties within the protein structure, were the analyses conducted.

### 2.8. The Identification and Selection Procedure of Binding Sites

Possible binding sites were identified using Discovery Studio by examining a series of physicochemical characteristics and searching for cavities. Nine possible binding sites were first identified. Surface area and volume of the pockets, hydrophobicity, electrostatic potential, the makeup of the amino acids in the surrounding area, and the possibility of ligand interactions were a few of the characteristics used to contrast these sites. Then, the optimal binding site was chosen for subsequent docking studies [17].

### 2.9. Preparation of Ligands and Molecular Docking of Neem Compounds

Because of the very well-documented anticancer properties and the wide range of bioactive chemicals present in neem extracts, neem (*A. indica*) compounds were chosen for docking studies [18]. A large library of reported neem compounds was compiled from various natural product databases and literature references. OpenBabel 3.1.0 was used to filter these compounds such that three-dimensional structures and appropriate protonation state at physiological pH could be more readily built.

### 2.10. Molecular Docking and Receptor Preparation

AutoDockTools was used for building the model of the MT—CO3 protein for molecular docking. Hydrogens to the polar atoms were added, Gasteiger charges were calculated, and a starting grid box centrally located in the binding site assigned was set up as part of this setup. Molecular docking was performed using AutoDock Vina [19].

### 2.11. Docking, Analysis, Visualization, and Interaction

Binding affinity scores generated through AutoDock Vina were used for the analysis of docking data. Correlation analysis of docking data to the MT—CO3 mean expression value was also carried out, as well as investigating certain protein–ligand interactions. The first five compounds were chosen for additional analysis and depiction. The protein-–ligand complexes of these high-scoring compounds were imaged, and interaction studies in close-up views, including π–π stacking, salt bridges, hydrogen bonding interactions, and hydrophobic interactions, were carried out. Two-dimensional interaction plots were drawn to describe the fine details of interactions between the neem compounds and MT—CO3 binding site residues. R software (version 4.1.0) and pertinent bioinformatics packages like DESeq2 and limma were utilized for statistical research. By determining the common substructure of the five compounds, scaffold weights and ratios were computed. Fragment efficiency was calculated by binding energy divided by the number of heavy atoms in the fragment, fragment efficiency was calculated. By removing the terminal rotatable bonds, the RDKit was used to compute rotatable bonds. The approach outlined by Bickerton et al. [20] was used to compute the quantitative determinant of drug-likeness (QED), taking into account rotatable bonds, TPSA, H-donors, H-acceptors, molecular weight, and LogP. Each property was normalized to a 0–1 scale and compared to known drug-like cutoffs, and normalized drug-like properties were computed.

### 2.12. Statistical Analysis

All statistical testing was conducted at a significance level of *p* < 0.05. Differential gene expression analysis used the Benjamini–Hochberg false discovery rate (FDR) adjustment for multiple testing corrections. Mann—Whitney U tests were used for non-parametric distributions of data

## 3. Results

**Figure 1:**
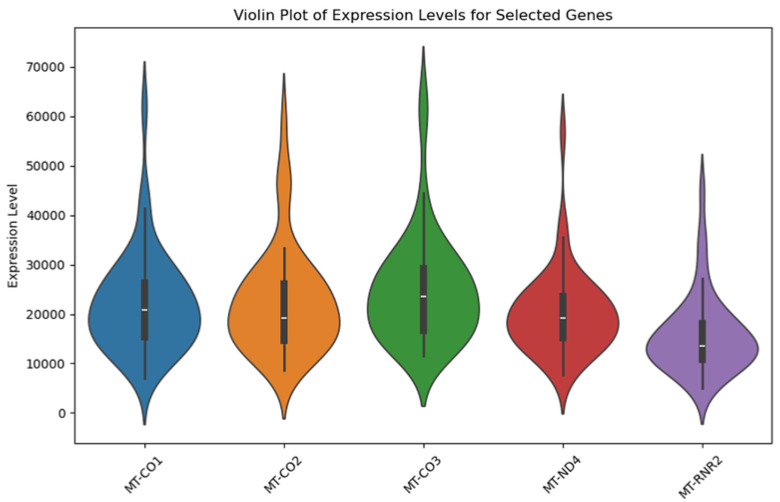
Violin plot illustrating the expression levels of the five most upregulated mitochondrial genes in breast cancer tissues relative to matched normal controls. The plot illustrates the differential expression of the genes MT—CO1, MT—CO2, MT—CO3, MT—ND4, and MT—RNR2. Each violin illustrates the probability density distribution of expression values, with broader sections signifying a greater likelihood of observing samples at that expression level. The thick black vertical line in the center indicates the median value, the thinner vertical black bar represents the interquartile range (25th to 75th percentile), and the thin black lines (whiskers) extend to the minimum and maximum values within 1.5 times the interquartile range. Expression levels are represented as transcripts per million (TPM) values.

**Figure 2:**
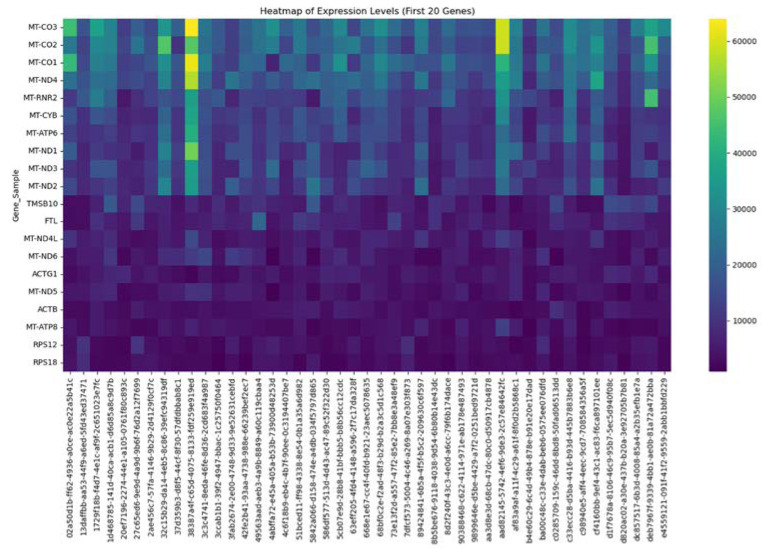
Heatmap of expression levels of the twenty most upregulated mitochondrial genes in breast cancer tissues relative to matched normal controls. Expression levels are represented as transcripts per million (TPM) values.

**Figure 3:**
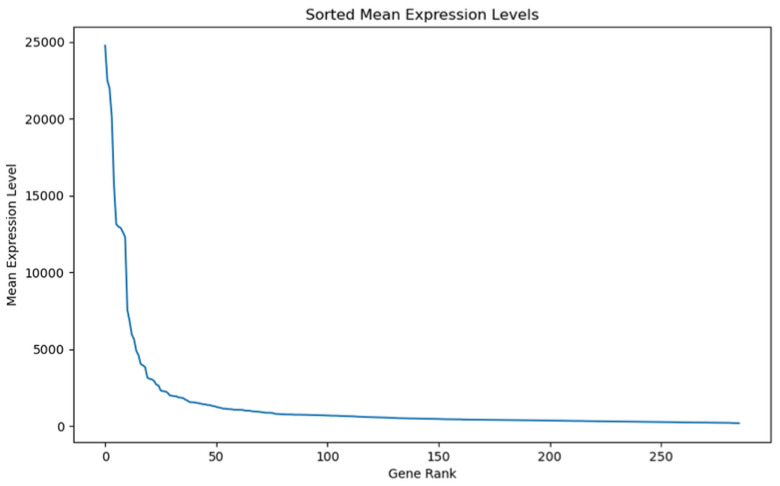
Gene rank vs. mean expression level. Expression levels are represented as transcripts per million (TPM) values.

**Figure 4:**
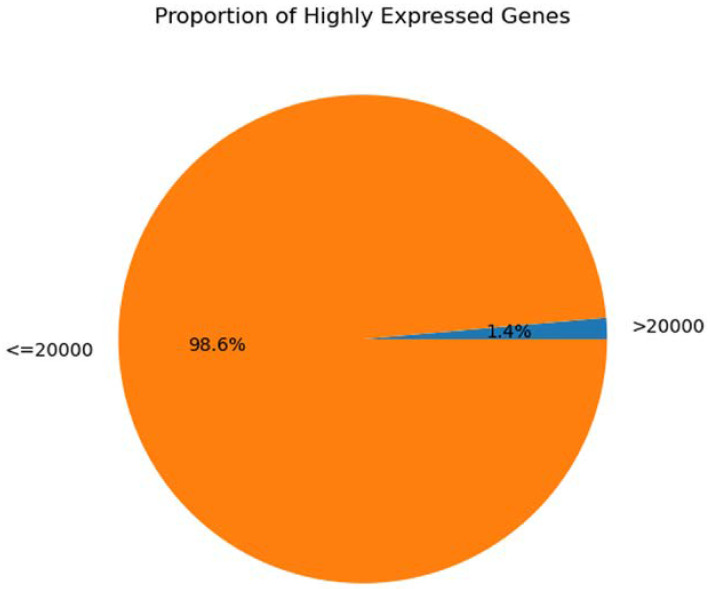
Proportion of highly expressed genes. Expression levels are represented as transcripts per million (TPM) values.

**Figure 5:**
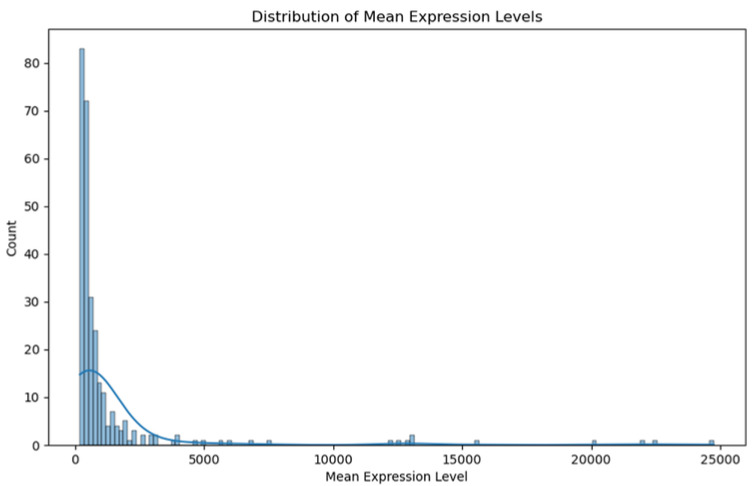
Distribution of mean expression level. Expression levels are represented as transcripts per million (TPM) values.

**Figure 6:**
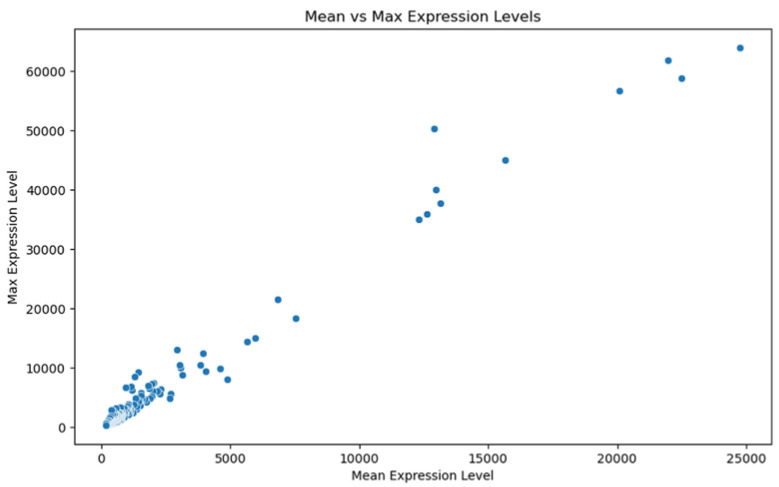
Scatter plot: mean vs. Max expression level. Expression levels are represented as transcripts per million (TPM) values.

**Figure 7:**
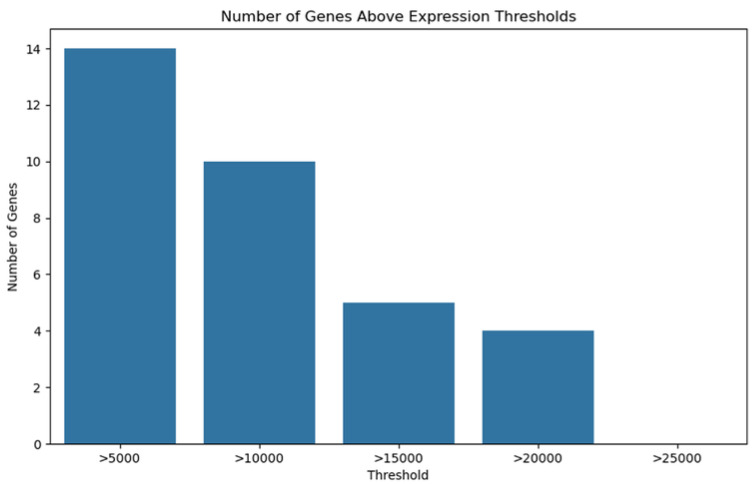
Number of genes above expression threshold. Expression levels are represented as transcripts per million (TPM) values.

**Figure 8:**
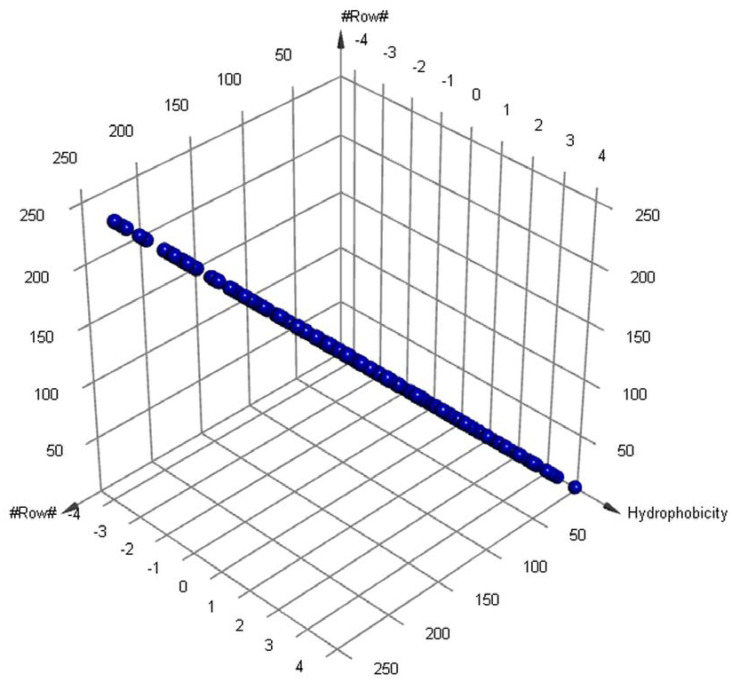
The 3D point plot of MT—CO3 modeled protein.

**Figure 9:**
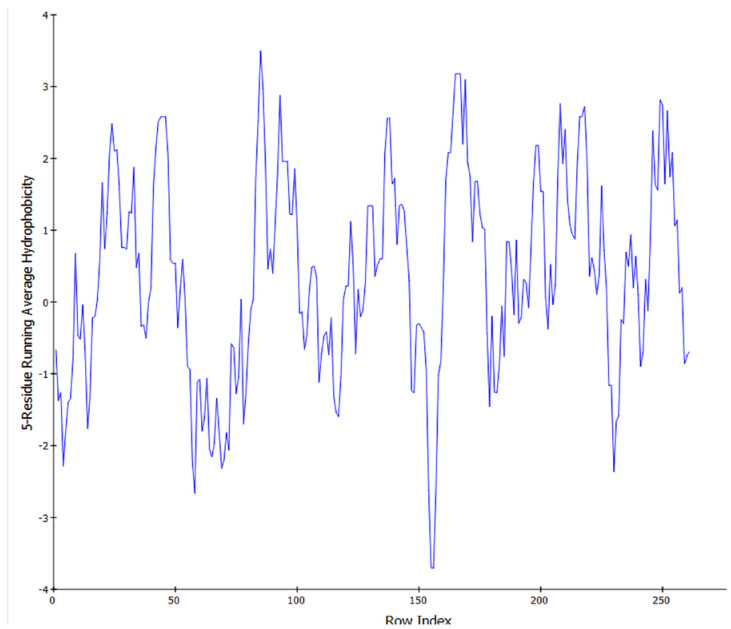
Hydrophobicity plot of MT—CO3 modeled protein.

**Figure 10:**
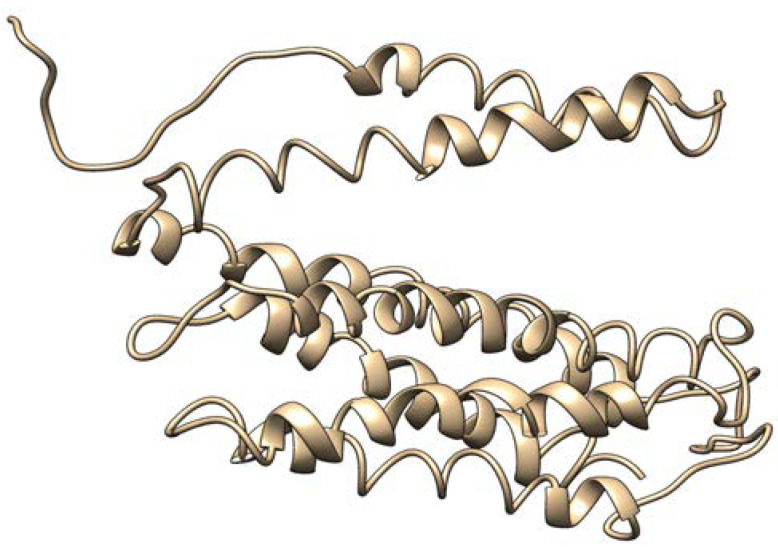
The 3D structure of the MT—CO3 modeled protein.

**Figure 11:**
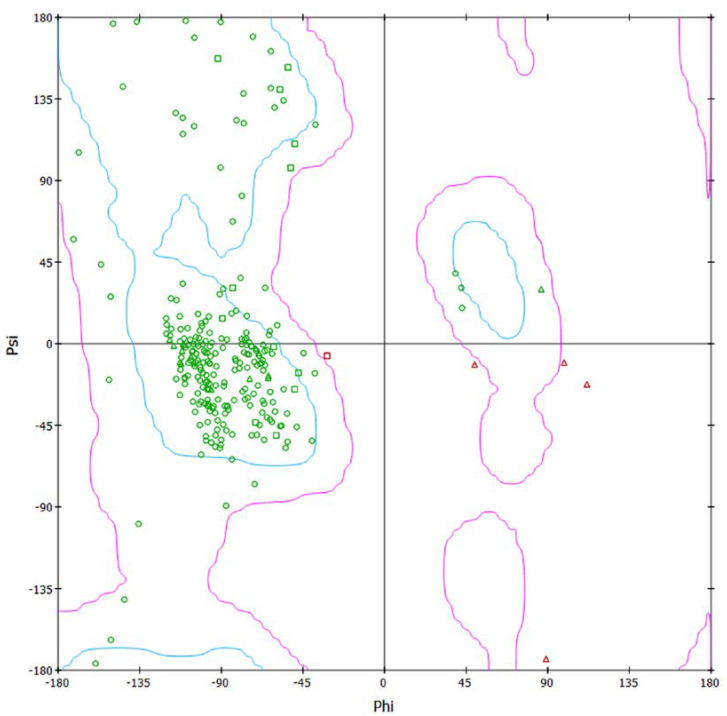
Ramachandran plot of the MT—CO3 modeled protein. The green and red symbols represent different amino acid residues plotted on the Ramachandran plot, which displays the phi (φ) and psi (ψ) torsion angles of the protein backbone. Specifically: green circles (○) represent residues in the “favored” regions of the Ramachandran plot, green squares (□) represent glycine residues in favored regions, red triangles (△) represent residues in “outlier” regions that deviate from preferred conformations, green triangles (△) represent proline residues in favored regions.

Our genomic and in silico analysis has shown strong evidence for the critical role of MT—CO3 in breast cancer development, as well as the discovery of possible neem-derived compounds as drug leads. The findings of this study can be distilled into three connected themes. MT—CO3 emerges as a critical figure in breast cancer pathogenesis. Based on our extensive investigation of gene expression values (expression levels are represented as transcripts per million (TPM), MT—CO3 is the most significantly overexpressed gene among breast cancer tissues. This outcome is especially notable considering the mitochondrial origin of MT—CO3 and its key function in the energy metabolism of the cell. Both the violin plot (Figure 1) and heatmap (Figure 2) obviously reflect MT—CO3 dominance with the highest median expression and most stable overexpression over a panel of breast cancer samples. The overall overexpression indicates the upregulation of MT—CO3 may be an inherent characteristic of breast cancer cells and not restricted to selected subtypes. Aside from confirming the significance of MT—CO3, our gene expression profile analysis (Figure 3, Figure 4, Figure 5, Figure 6 and Figure 7) consistently positioned MT—CO3 at the extreme end of a number of measures: it had the highest mean expression level (Figure 3), contributed disproportionately to overall transcriptome composition (Figure 4), and exhibited extremely high expression levels in samples (Figure 5, Figure 6 and Figure 7). This repeated overexpression by multiple analytical methods strongly suggests that the overexpression of MT—CO3 is not a statistical fluke but a robust biological phenomenon associated with breast cancer. Structural insights into MT—CO3: A potential therapeutic target since there has been extreme overexpression of MT—CO3, we used extensive protein modeling to provide a basis for the understanding of structural elements that will inform therapeutic approaches. Our search revealed several principal features: 3D point plot and hydrophobicity analysis (Figure 8 and Figure 9) revealed a substantial hydrophobic pocket, which is potentially essential for protein-protein interaction or may serve as a putative small molecule-binding site. The global three-dimensional topology (Figure 10) displayed a knotted folding motif that is typical of mitochondrial membrane proteins, with potential transmembrane regions that could be drug-targetable. The Ramachandran plot (Figure 11) confirmed the higher quality of our model, thereby lending strength to subsequent docking investigations. These structural data are particularly significant since experimental structures of proteins from mitochondrial DNA continue to be in short supply. Our model offers a robust platform for structure-based drug design strategies to address MT—CO3.

### Neem-Derived Compounds as Potential Inhibitors of MT—CO3

**Figure 12:**
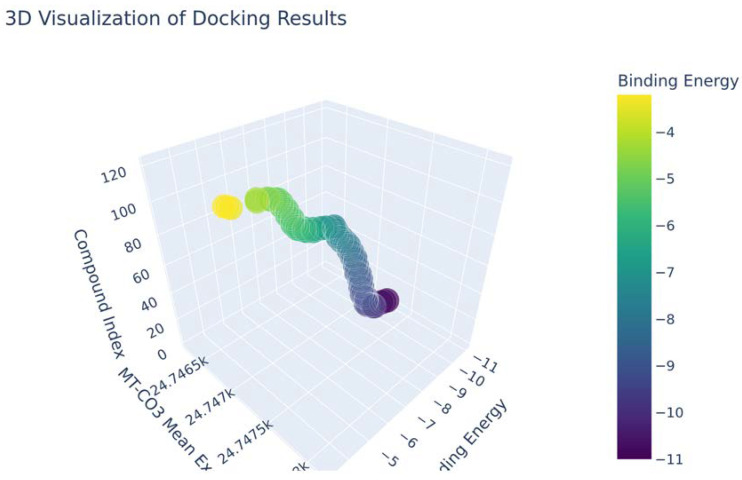
The 3D Neem _compounds docking results.

**Figure 13:**
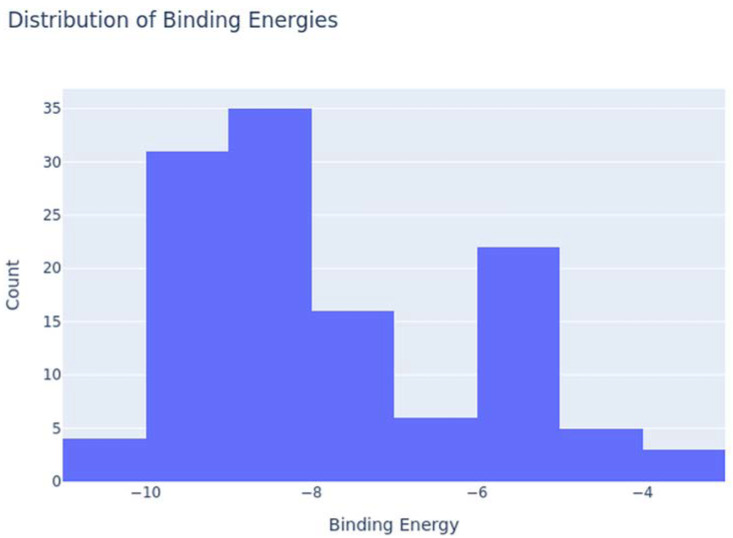
Neem compound binding energy distribution on MT—CO3 modeled proteins.

**Figure 14:**
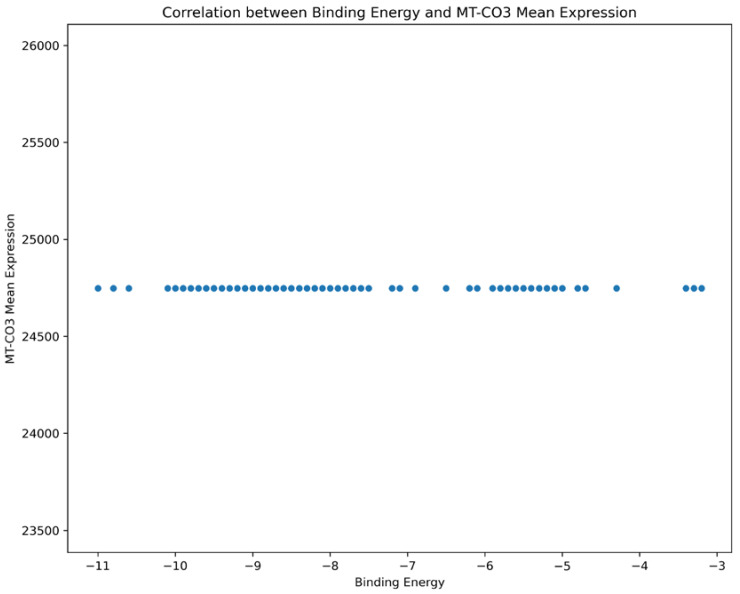
Neem compounds clustering throughout binding energy ranges (MT—CO3 mean expression vs. neem compound binding energy clustering). Every dot represents a separate compound screened versus the MT—CO3 protein structure. The clustering pattern demonstrates energy levels at which numerous compounds share a comparable binding affinity, which shows specific structural elements favorable to MT—CO3 interaction.

**Figure 15:**
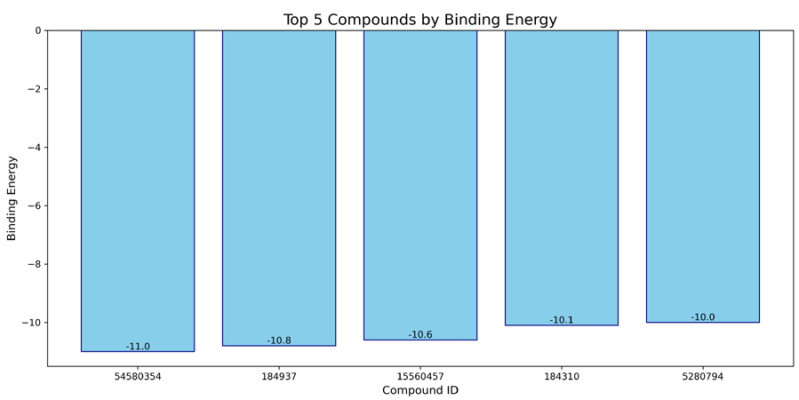
Top five neem compounds identified on the MT—CO3 modelled proteins. 54580354: 7-benzoylnimbocinol, 184937: nimolicinol, 15560457: melianodiol, 184310: isonimocinolide 5280794: stigmasterol.

**Figure 16:**
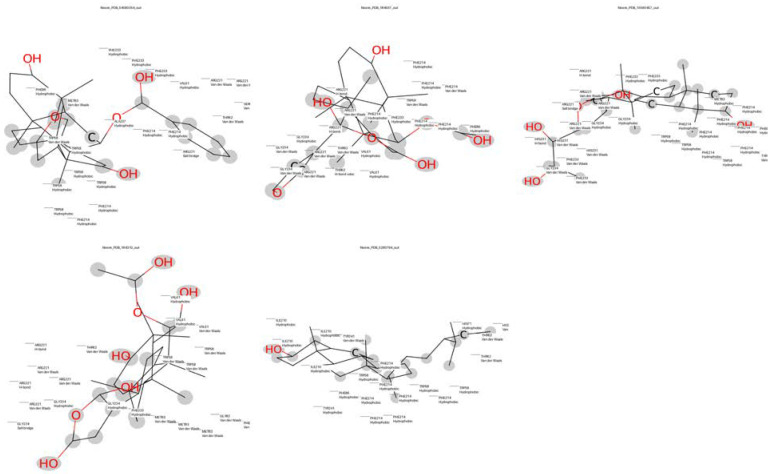
Top five neem compounds ligand interaction with the MT—CO3 modeled proteins. Neem_PDB_54580354_out: 7-benzoylnimbocinol, Neem_PDB_184937_out: nimolicinol, Neem_PDB_15560457_out: melianodiol, Neem_PDB_184310_out: isonimocinolide Neem_PDB_5280794: stigmasterol.

**Figure 17:**
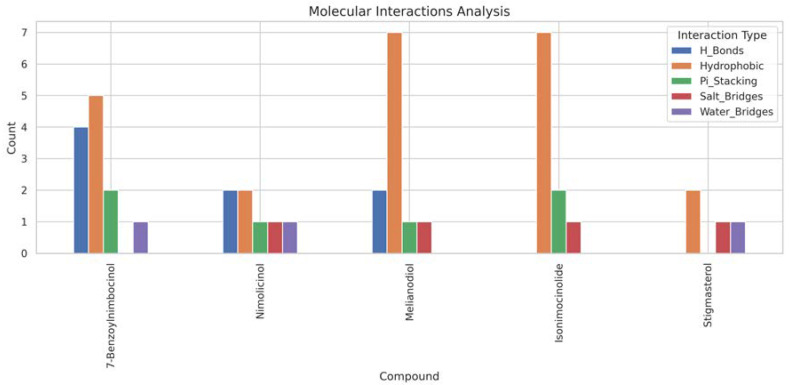
The pattern of binding of topmost five neem compounds on MT—CO3 proteins.

**Figure 18:**
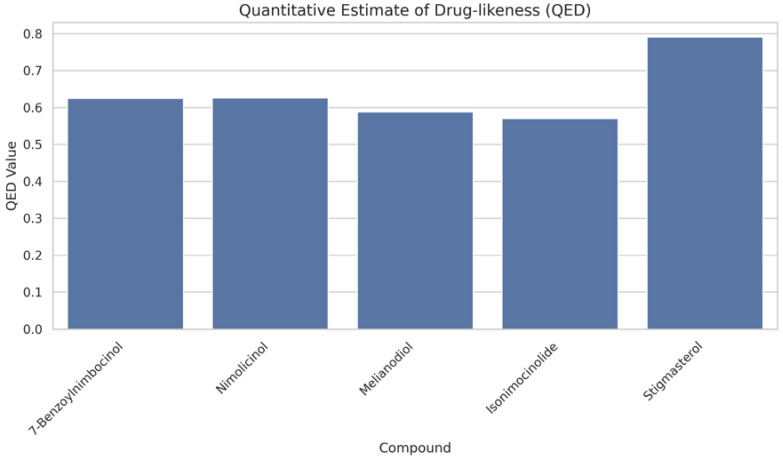
Quantitative estimate of drug-likeness (QED) analysis of the topmost five neem compounds.

**Figure 19:**
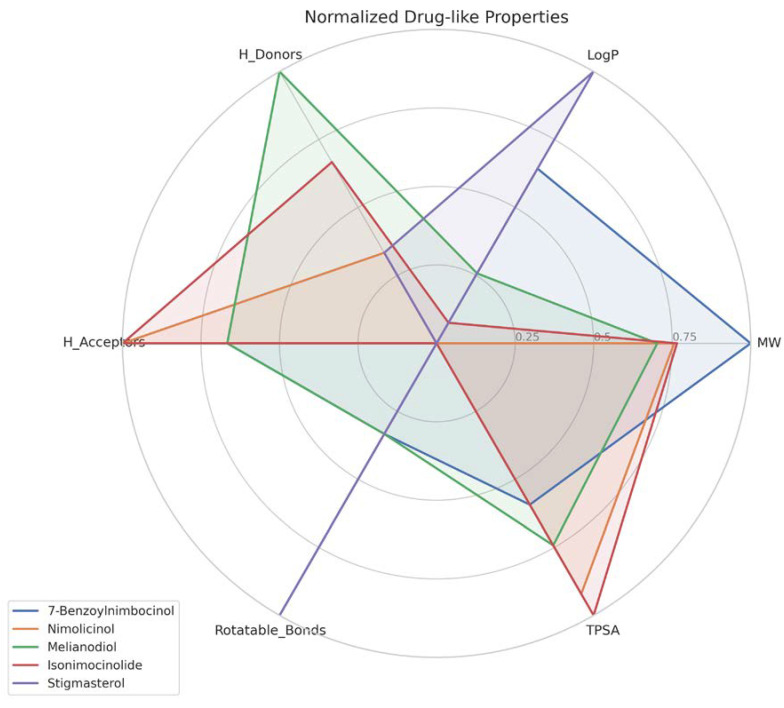
Drug-like features radar plot of the topmost five neem compounds on MT—CO3 proteins.

Based on our structural model, we conducted a comprehensive docking study with neem-derived compounds that possess diverse bioactivities. This study yielded several interesting findings. The three-dimensional docking result (Figure 12) indicated that multiple neem compounds interacted in a comparable manner with a specific pocket on MT—CO3, and this implies a potential mechanism for modulating its activity. The distribution of binding energies (Figure 13) showed the count of the neem compounds binding to MT—CO3. Figure 14 corroborates this and shows the MT—CO3 protein target clustering of the neem compounds. The compounds display a broad spectrum of binding affinities (−3 to −11 kcal/mol), with some clustering within specific energy windows. The variation of binding energy accounts for the structural heterogeneity of the compounds and their interaction capacity with the MT—CO3 binding pocket, yielding valuable information for the choice and optimization of lead compounds. The five leading compounds (Figure 15) with excellent binding profiles were identified from our evaluation: 7-benzoylnimbocinol, nimolicinol, melianodiol, isonimocinolide, and stigmasterol. These compounds had interactions with a network of interactions with MT—CO3, including hydrogen bonds, hydrophobic interactions, and potential π-–π stacking (Figure 16). The diversity of these interactions indicates a robust binding mode that would be capable of efficiently modulating MT—CO3 activity. In conclusion, these results not only identify MT—CO3 as a novel therapeutic target in breast cancer but also designate some neem-derived compounds as potential drug candidates. The observed correlation between MT—CO3 expression levels and binding affinity is particularly significant, as it suggests the potential for a targeted therapeutic intervention that would selectively impact cancer cells while sparing normal tissues. The molecular interaction pattern revealed hydrophobic is the major mode of interaction for all compounds, and melianodiol and isonimocinolide have the highest frequencies (*n* = 7) (Figure 17). 7-Benzoylnimbocinol exhibited the most diverse pattern of interaction with the highest hydrogen bonding potential (*n* = 4) complemented with hydrophobic (*n* = 5) and π-stacking (*n* = 2) interactions. Quantitative Estimate of Drug-likeness (QED) analysis showed that stigmasterol possesses greater drug-like characteristics (QED = 0.79) when compared to the other compounds (QED range: 0.56–0.62) (Figure 18). A comparison of normalized drug-like property radar plots revealed that while stigmasterol is better in lipophilicity (LogP) and molecular weight properties but bad in hydrogen bond donors, melianodiol possesses a more balanced physicochemical profile with the best hydrogen donor capability (Figure 19). Fragment contribution analysis also reveals differential scaffold contribution to molecular properties across the compound set. The scaffold percentage of total molecular weight is highly variable between compounds and affects binding energy efficiency. Energy per heavy atom calculations show that compounds with ideal fragment efficiency have better binding properties independent of molecular weight variation (Table 1).

## 4. Discussion

Strong evidence for the essential role of MT—CO3 in the pathophysiology of breast cancer has been discovered by our integrative genomic and in silico studies, which have also revealed potential chemicals from neem as potential treatment options. This study started with a thorough examination of 59,428 genes associated with breast cancer. To provide a reproducible and consistent dataset to study further, we narrowed our focus to 286 genes by imposing strict filtering based on persistent expression in 42 samples. The prominent overexpression of MT—CO3 in our revised gene set represents a shift in paradigm in breast cancer biology. One of the mitochondrial electron transport chain components that has not been heavily targeted in cancer research is subunit III of cytochrome c oxidase (complex IV), which is coded by MT—CO3. A straightforward reprogramming of cellular energetics in cancer cells is indicated by its chronic elevation in a sequence of breast cancer samples. This is in line with Chen et al. [21] emphasis on the increasing recognition of mitochondrial dysfunction in cancer. While the nuclear-encoded mitochondrial genes have received considerable attention, our study emphasizes the critical function of genes encoded by the very mitochondria themselves. Consistent with the “reverse Warburg effect” foretold by Jaworska et al. [22], overexpression of MT—CO3 may act as compensation to ensure provision for the extensive energy needs of vigorously growing cancer cells. Additionally, overexpression of MT—CO3 may be one of the factors leading to the shifted redox state found in various types of cancer. Since cytochrome c oxidase is one of the main sources of reactive oxygen species (ROS), MT—CO3 overexpression would lead to more oxidative stress, a typical property of cancer cells [23]. To construct a feed-forward loop of malignancy transformation, the resulting oxidative stress may augment genomic instability and fuel tumor progression. We fill a crucial gap in the literature by presenting the first complete structural picture of MT—CO3 with our protein modeling investigations. Most remarkable is the finding of an extensive hydrophobic pocket in the protein structure. In cardiolipin, a cytochrome c oxidase assembly and activity lipid, the pocket might be utilized as a binding site [24]. If this connection is severed, it can affect MT—CO3 activity and, subsequently, cancer cell mitochondrial function. Drug design is both challenged and enabled by the intricate folding structures present in our model, which are characteristic of proteins in the mitochondrial membrane. The transmembrane segments hold the promise of binding sites for peptidomimetics or other therapeutic options like stapled peptides, although potentially challenging to address using traditional small molecules, as suggested by Azzarito et al. [25]. Through the clarification of intricate interactions between neem-derived compounds and the MT—CO3 protein, our docking studies have illuminated considerably new information regarding potential mechanisms of action. These interactions, their effects on protein function, and their therapeutic implications are all explored in great detail in this paper. The complex interactions of neem compounds with a protein model linked to MT—CO3 are explored in this computational study, offering new insights into possible therapeutic interventions for breast cancer. The five structurally diverse neem compounds—7-benzoylnimbocinol, nimolicinol, melianodiol, isonimocinolide, and stigmasterol—have complex binding moieties with substantial influence on cancer cell and mitochondria metabolism [26]. Phe233, Phe214, Arg221, Met83, and Trp58 are the residues that 7-benzoylnimbocinol binds with. Hydrogen bonding with Arg221 improves binding specificity, while π–π stacking interactions between its phenyl rings and Phe233/Phe214 furnish a solid anchoring point [27]. A close fit in a well-defined binding pocket is also indicated by hydrophobic contacts with Trp58 and Met83, which contribute to the stability of the ligand–protein complex [28]. F214, F86, V61, R221, and W58 are all occupied by nimolicinol. Overlapping contacts by 7-benzoylnimbocinol (W58, F214, and R221) indicate that these limonoids share a common binding site. The two non-polar segments of nimolicinol are located in a hydrophobic subpocket that is supported by Val61 and Phe86 [29]. Melianodiol is bound to Phe214, His231, Met83, Gly234, Phe233, and Arg221. The presence of His231 and Gly234 suggests a flexible region that adjusts to this triterpenoid compound [30]. His231 is responsible for regulating the catalytic process of the protein, most probably by forming hydrogen bonds with melianodiol hydroxyl groups [31]. In its interaction with Val61, Trp58, Thr62, Arg221, and Gly234, isonimocinolide forms polar as well as hydrophobic interactions. A hydrophobic cleft is formed by Trp58 and Val61, and hydrogen bonding is formed by Thr62 with oxygen-containing groups of the compound [32]. Stigmasterol’s sterol backbone is echoed in its behavior with Phe214, Ile210, Tyr241, Trp58, His71, and Thr62. Tyr241 and Ile210 hydrophobically interact with the steroid nucleus, with His71 participating through hydrogen bonding with the hydroxyl group [33]. Its interaction profiles provide an assortment of salient points. Its structurally variant compounds are indicated by a conserved binding site, depicted by the repeated appearance of Phe214, Arg221, and Trp58 among varied ligands. This variation could prove helpful in drug resistance in cancer treatment [34]. The diverse interaction involving hydrophobic (Val, Ile, and Met), charged (Arg and His), and aromatic (Phe, Trp, and Tyr) residues expresses a refined interaction between hydrophobic, π-π stacking, and hydrogen bonding. High affinity and specificity are enhanced by this binding mode [35]. Gly and Thr residues point towards flexible areas being implicated, meaning that such molecules are able to induce conformational changes leading to allosteric control of protein function [36]. Interaction with His231 and Arg221 might have direct effects on mitochondrial activity through electron transfer or proton translocation channels important to cytochrome c oxidase activity [37]. The molecular interaction profiles of this research give critical new insights into the binding modes of the top five molecules under consideration. Similarly to all other studies on its bioactivity, the high hydrogen-bonding capacity of 7-benzoylnimbocinol offers scope for target selectivity [27]. Since melianodiol and isonomocinolide have been found to possess the potential for penetration through lipophilic matrices, their affinity for hydrophobic interactions can be attributed to their reported biological activity [32]. Sterols are likely to have desirable physicochemical profiles in drug development, according to Qi et al.’s report [33], as indicated by stigmasterol’s superior drug-likeness score (QED = 0.79). The report from the radar plot also shows how stigmasterol’s highly balanced profile in a number of factors adds up to its high QED score, particularly its ideal LogP value and rotational bond count. The observed anti-inflammatory and anticancer activities can be explained by this profile [34]. Fragment contribution analysis can prove to be of great value in future optimization plans. Fragment efficiency and scaffold size are inversely related, meaning that functionals on small scaffolds are more likely to yield good binders. Earlier literature has described this phenomenon in natural product compound optimization [38]. Each heavy atom binding energy is comparable for the compounds (−0.289 to −0.333 kcal/mol), implying that identical energetic principles apply to molecular recognition despite structural differences. The 14 scaffold atoms shared among the five best-ranking compounds are an indication of a potential pharmacophore that can be applied in scaffold hopping methods. Furthermore, the relatively stable energy per heavy atom is an indication that in the event the variable segments are targeted, especially with changes, and the central scaffold is left intact, binding affinity increases may follow. To guide the development of more active and selective analogs, the different interaction profiles of these structurally diverse neem compounds are useful for structure-activity relationship studies [38]. Neem compound interactions with the MT—CO3 protein unveil a mechanism of cancer cell metabolism modulation through modification of mitochondrial function, and these findings are of special relevance in breast cancer treatment [39]. Most agree that mitochondrial dysfunction is among the cancer signatures and, therefore, a suitable target for treatment [40]. Besides, the heterogeneity of interaction profiles implies that these compounds could target cancer cells through various mechanisms, which would make them capable of breaking the barriers presented by adaptive resistance and tumor heterogeneity, which have a tendency to limit therapies with narrow targets [41]. In the development of new drugs, the large body of interaction data provides a foundation for either the design of new synthetic mimics of higher potency and specificity or the optimization of these new compounds based on their structure. The results from this study underline the importance of natural products in disease management [42,43,44,45]. While this research provides useful insights pertaining to MT—CO3 as a drug target, there are limitations that must be addressed. Our in silico modeling strategy, as thorough as it is, could be complemented through the experimental confirmation of binding affinities. Additionally, the current analysis overlooks potential synergistic effects of drugs along with potential toxicity concerns in vivo. Future work must include these points through experimental confirmation and mixtures of the compounds found.

## 5. Conclusions

In conclusion, the current research points towards MT—CO3 as a novel drug target for breast cancer and offers a rationale for structure-based drug discovery from natural products. By integrating ancient intuition with contemporary genomics and computational biology, we are poised to explore new frontiers in the war against breast cancer. In the future, it will be required to verify these in silico findings through longer in vitro and in vivo studies to make possible the development of a new class of cancer drugs targeted against mitochondria. Other than its theoretical implications, this study has direct practical value in drug discovery by calling attention to specific molecular backbones and binding sites that can guide medicinal chemistry efforts toward more evolved MT—CO3 inhibitors with enhanced potency and selectivity. These discoveries have the potential to fast-track the translation of neem compounds to clinical trials for breast cancer treatment.

## Figures and Tables

**Table 1:** Fragment contribution analysis of the topmost five neem compounds on MT—CO3 proteins.

Compound	Molecular Weight	Scaffold Weight	Scaffold Proportion	Rotatable Bonds	Binding Energy (kcal/mol)	Energy per Heavy Atom	Common Scaffold Atoms	Fragment Efficiency
7-Benzoylnimbocinol	512.26	442.18	0.86	3	−11.00	−0.29	14	−0.03
Nimolicinol	482.23	338.15	0.70	2	−10.80	−0.31	14	−0.03
Melianodiol	488.35	314.22	0.64	3	−10.60	−0.30	14	−0.03
Isonimocinolide	484.25	324.17	0.67	2	−10.10	−0.29	14	−0.03
Stigmasterol	412.37	230.20	0.56	5	−10.00	−0.33	14	−0.04

## Data Availability

The original contributions presented in this study are included in the article/supplementary material. Further inquiries can be directed to the corresponding author.

## Supplementary Materials

The following supporting information can be downloaded at: https://www.mdpi.com/article/10.3390/genes16050546/s1, Figure S1: 3d_docking_results.html; Figure S2: binding_energy_distribution.html; Figure S3: binding_expression_correlation.html.

## Abbreviations

TCGA-BRCA	The Cancer Genome Atlas—Breast Cancer
MT—CO3	mitochondrially encoded cytochrome C oxidase III
RNA	ribonucleic acid
GDC	genomic data commons
PDB	protein data bank
2D	two-dimensional
3D	three-dimensional
MMFF94	Merck molecular force field 1994
